# TC-hunter: identification of the insertion site of a transgenic gene within the host genome

**DOI:** 10.1186/s12864-022-08376-0

**Published:** 2022-02-20

**Authors:** Vanja Börjesson, Angela Martinez-Monleon, Susanne Fransson, Per Kogner, John Inge Johnsen, Jelena Milosevic, Marcela Dávila López

**Affiliations:** 1 Bioinformatics Core Facility, Sahlgrenska Academy, University of Gothenburg, Gothenburg, Sweden; 2 Department of Laboratory Medicine, Institute of Biomedicine, Sahlgrenska Academy, University of Gothenburg, Gothenburg, Sweden; 3 Childhood Cancer Research Unit, Department of Women’s and Children’s Health, Karolinska Institutet, Stockholm, Sweden; 4 Center for Regenerative Medicine, Massachusetts General Hospital, Boston, MA 02114 USA

**Keywords:** Next-generation sequencing, Transgenic insertion site, PPM1D, Chimeric reads, Discordant read pairs, Transgenic organisms

## Abstract

**Background:**

Transgenic animal models are crucial for the study of gene function and disease, and are widely utilized in basic biological research, agriculture and pharma industries. Since the current methods for generating transgenic animals result in the random integration of the transgene under study, the phenotype may be compromised due to disruption of known genes or regulatory regions. Unfortunately, most of the tools that predict transgene insertion sites from high-throughput data are not publicly available or not properly maintained.

**Results:**

We implemented TC-hunter, Transgene-Construct hunter, an open tool that identifies transgene insertion sites and provides simple reports and visualization aids. It relies on common tools used in the analysis of high-throughput data and makes use of chimeric reads and discordant read pairs to identify and support the transgenic insertion site. To demonstrate its applicability, we applied TC-hunter to four transgenic mice samples harboring the human *PPM1D* gene, a model used in the study of malignant tumor development. We identified the transgenic insertion site in each sample and experimentally validated them with Touchdown-polymerase chain reaction followed by Sanger sequencing.

**Conclusions:**

TC-hunter is an accessible bioinformatics tool that can automatically identify transgene insertion sites from DNA sequencing data with high sensitivity (98%) and precision (92.45%). TC-hunter is a valuable tool that can aid in evaluating any potential phenotypic complications due to the random integration of the transgene and can be accessed at https://github.com/bcfgothenburg/SSF.

**Supplementary Information:**

The online version contains supplementary material available at 10.1186/s12864-022-08376-0.

## Background

Transgenesis is one of the major tools to study gene expression and function [1], it is a process where a gene (transgene) is introduced from one organism into the genome of another organism. These transgenic organisms, including plants, animals, bacteria and viruses [2], have had a considerable impact on biomedical research and human welfare. Common examples are the improvement of livestock and crops’ quality, the increase in production of medically useful substances via “pharming”, safer xenotransplantation, and the furtherance in the study of gene function and therapy of diverse human diseases [1–5].

There are several methods to generate transgenic animals [1], from which pronuclear injection and retroviral transduction result in the random integration of the transgene in the host [6]. Transgene integration may produce transgene silencing or altered transgene expression if the integration targets the heterochromatin or euchromatin, respectively, or it can result in the inactivation of a disrupted endogenous gene ([7, 8] and references therein).

To identify the insertion site (IS) of the integration event Polymerase Chain Reaction (PCR)-based techniques are often used. However, the presence of multiple integration events or the use of large transgenes, may question the reliability of the detection method [8]. Nevertheless, the location of the integration provides relevant information such as the prediction of potential phenotypic complications [9] and rearrangements of the transgene or the host genome at the IS. This could serve as a filtering process, preventing the use of any transgenic organism with an unintentional activation of oncogenes.

With the advent of sequencing data, today it is more cost effective to identify the transgene insertion site (TIS) with *in-silico* routine analysis. There have been several efforts to predict TIS using next generation sequencing (NGS) data that rely on chimeric reads and discordant read pairs [8–12], i.e. pairs that map in non-canonical ways. Unfortunately, some of these methods were developed as in-house algorithms and are not publicly available. Others are not compiled as a single workflow or lack documentation for a proper installation, making its application cumbersome and time consuming (Supplementary Table S1).

Therefore, we present TC-hunter, Transgene-Construct hunter, a bioinformatics tool that predicts the IS of a given transgene construct in its host genome, given a sufficiently contiguous reference assembly. TC-hunter is easy to install, well documented and it makes use of common bioinformatics software applied in the analysis of NGS data. It creates simple reports and visualizations that can be used to assess the IS of the construct and guide its experimental validation. We demonstrate its applicability by identifying and validating the IS of a p53-regulated gene, *PPM1D*, in a transgenic mouse model used in the study of malignant tumor development [13].

## Implementation

### TC-hunter overview

**Fig. 1:**
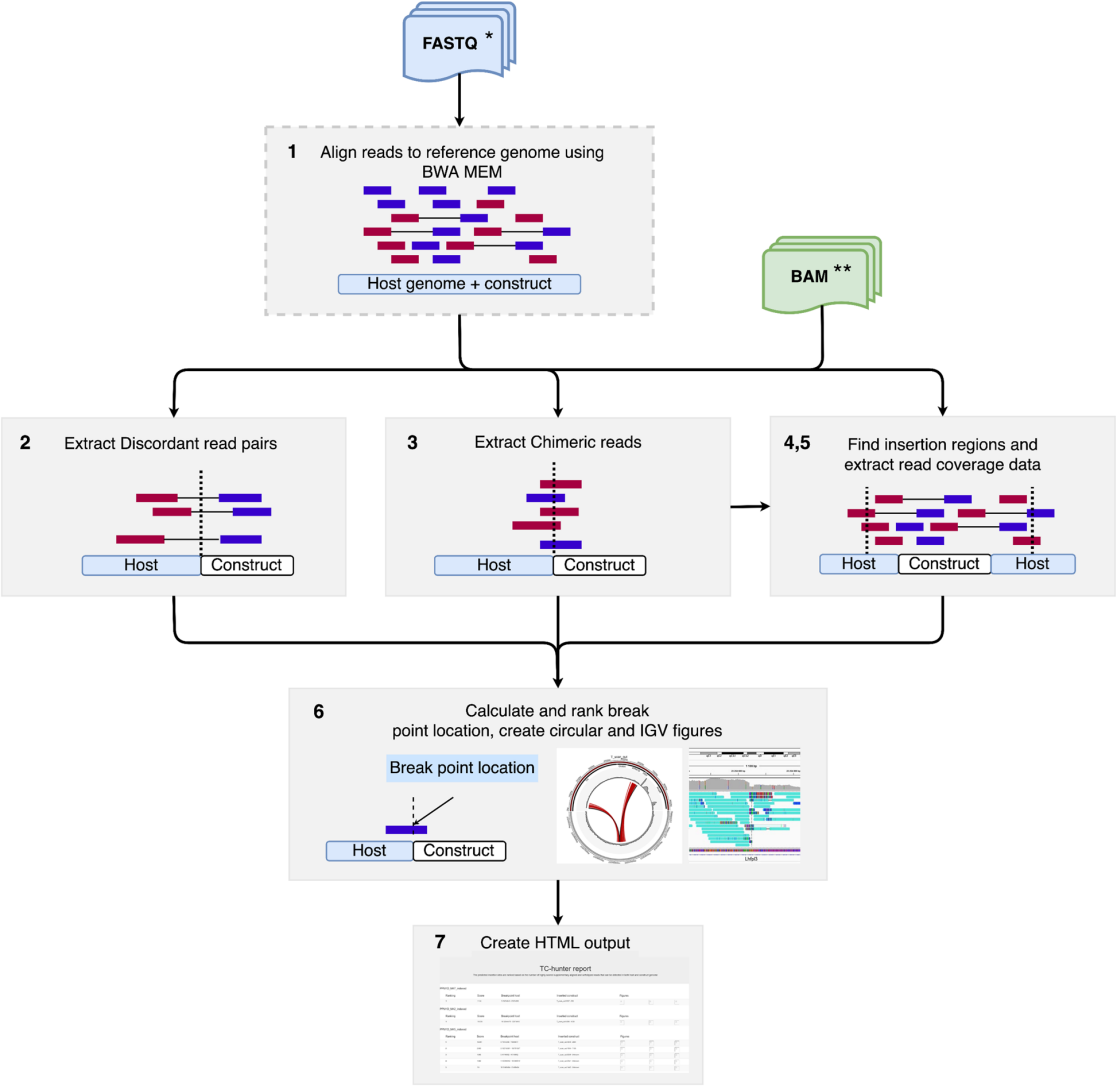
TC-hunter workflow and subprocesses. TC-hunter can be run with either fastq files (blue) or BAM files (green). * If fastq files are the input, the construct and host reference files are needed as well as the genomic annotation of the construct. ** If BAM files are the input, the alignment should be done against a joint genome (construct + host references), a genomic annotation file of the construct is also needed. Reads are depicted as red (forward reads) and blue rectangles (reverse reads), where a connecting line indicates both reads are paired. 1) The configuration file will dictate if fastq files are to be aligned to a composite reference genome (host genome + construct sequence). 2) TC-hunter extracts information about discordant read pairs (those where one read is aligned to the host and the other read is aligned to the construct) and 3) chimeric reads (those where a single read aligns to both the host and the construct). 4) Then, this information is used to detect the transgenic insertion region(s) and 5) to extract coverage data. 6) Next, TC-hunter determines the break point location of the transgenic insertion site and ranks the results according to coverage evidence. Finally, it generates visualization aids and a summary report 7) for further evaluation of the results

TC-hunter is a Nextflow [14] pipeline that scans NGS data to report predicted TIS within a sufficiently contiguous host genome making use of chimeric reads and discordant read pairs. A chimeric read is when a single read aligns to two distant genomic regions, e.g. one part of the read aligns to the transgenic construct and another part aligns to the host genome (Fig. 1, box 3). On the other hand, discordant read pairs are those whose alignment to the reference genome have a distance and/or orientation different from expected, e.g. when one entire read aligns to the host genome while the read pair aligns to the construct (Fig. 1, box 2). TC-hunter generates a summary report including all chromosomes (scaffolds or contigs) that contain at least one chimeric read. For each one of these candidates, an evidence-based score is reported (see under *Pipeline description*), as well as the corresponding circular graphical representation(s) and alignment snapshots, which facilitate the interpretation and filtering of the candidates. TC-hunter requires i) the construct sequence (fasta format), ii) the reference genome (fasta format), iii) the genomic annotation of the construct (BED format) and iv) the sequencing data (paired end fastq files or bam files aligned to the host and the construct sequence). Several samples can be run in the same analysis and the pipeline will automatically parallelize the jobs based on the resources available.

### Pipeline description

TC-hunter is an assembly of several scripts and tools scanning for insertion site(s) of a transgenic sequence within a host genome, and it generates graphical representations of the predicted TIS and their supporting data. The overall workflow consists of setting up a configuration file, an optional alignment step (if fastq files are used), data extraction (chimeric reads, discordant read pairs, read coverage) and processing (TIS detection), results visualization and summary reporting. Figure 1 illustrates how these steps are interconnected through channels that are handled in a main Nextflow script allowing for parallelization of data, scripts and tools. When analyzing several samples, the results are presented in one joint report. Moreover, TC-hunter can be resumed at any stage since Nextflow provides this capability.

TC-hunter is an open source pipeline available on GitHub (https://github.com/bcfgothenburg/SSF) and it requires the installation of the following tools: R 3.5 or higher [15], python 2.7 [16], samtools 1.10 [17], Nextflow 19.01.0 [14] and BWA 0.7 [18]. The pipeline has been validated with (but is not restricted to) these versions. Moreover, the tool includes an yml file for the quick installation of these necessary third-party tools using conda [19] environments. Detailed information of each step can be found at the GitHub page.

### Configuration file setup

TC-hunter supports two different input files; 1) raw fastq files, or 2) bam files already aligned to the construct and the host reference genome. The pipeline requires a configuration file containing the path to reference files (host and construct fasta files), sample fastq files or bam file(s) and the working directory. A text file with the construct metadata is also needed in order to add the corresponding annotation when generating the circular visualization(s).

### Fastq files alignment

When running with fastq files as input, as first step, TC-hunter creates a concatenated reference file containing both host and construct sequences. Then, the joint genome is indexed with BWA index using default parameters (Fig. 1, box 1). The fastq files are then aligned to this reference genome using the local aligner BWA MEM with default settings. The minimum score to output a mapping read is 30 and only primary alignments are considered. TC-hunter uses the insert size distribution as calculated by BWA MEM, which is inferred from a batch of the total sample reads. The BWA MEM algorithm allows split alignments and thus, the identification of chimeric reads. During the alignment, these chimeric reads undergo a soft-clipping step, annotating the bases that do not align to the host genome (or the construct) which aids TC-hunter in their extraction. samtools is used to sort and index the aligned BAM file(s). The user can specify the number of threads to use for BWA MEM in the configuration file.

### Chimeric reads and discordant read pairs extraction

In an alignment file, the CIGAR string is a field containing a compressed representation of how reads are aligned to the reference genome. TC-hunter makes use of the CIGAR string to extract all chimeric reads and stores their alignment coordinates in a text file (Fig. 1, box 2). The mapping quality threshold for the extraction of chimeric reads can be specified in the configuration file (default is 30).

TC-hunter scans the aligned bam file for discordant read pairs and extracts read pairs where one of the reads aligns to the construct (Fig. 1, box 3). These discordant reads are saved as an alignment file for downstream evaluation.

#### Transgenic insertion and break point sites detection

TC-hunter uses the chimeric reads to delimit the candidate region, where the outer reads define the position of the TIS (Fig. 1, box 4). Read coverage data is extracted and stored to be used in the visualization step (Fig. 1, box 5). To provide the exact location of the breakpoint (or junction) TC-hunter parses the CIGAR string of the chimeric reads (Fig. 1, box 6).

A prediction score is calculated based on the number of chimeric reads and discordant read pairs that support each IS for each candidate. This score is used to rank the detected IS and is calculated as follows:

1. prediction score = number of chimeric reads + (number of discordant read pairs / 1000)

Discordant read pairs cover a wider region and tend to be more abundant than the number of chimeric reads. Moreover, chimeric reads give a more precise location of where the insertion has taken place due to having reads split over the breaking point, and will therefore be weighted 1000 time higher than the discordant read pairs.

### Results visualization

For every predicted insertion site, a circular plot of the breakpoint positions in the host genome is generated by using the circlize [20], dplyr [21] and data.table [22] R libraries (Fig. 1, box 6). For this purpose, TC-hunter generates two datafiles, one containing the genomic information (karyotype) and the other with the data points (histogram). The plot includes all chimeric reads supporting the insertion site on the same chromosome and the discordant read pairs within 5000 bp up- or down-stream of the breakpoint positions. The plot also includes a histogram of read coverage over these regions as well as the construct metadata including its genomic annotation.

The Integrative Genomics Viewer [23] (IGV) is used to create two additional figures to visualize the reads mapped to the reference genome. The first figure represents the reads covering the entire region of the predicted IS in the host genome, while the second shows a zoomed version of the IS (± 4600 bp). If TC-hunter is run on a server that does not have access to the GUI (graphical user interface), there is an option to generate these figures from the command line with the.bat files that TC-hunter generates. Parameters of the figures can be modified by the user directly within the.bat files.

### Summary reporting

For each sample, TC-hunter reports all predicted insertion sites together with their corresponding graphical representations in a collective html file (Fig. 1, box 7). All sites per sample are ordered based on the prediction score, with the highest scored site first. If specified, TC-hunter will create a second html report containing the Nextflow workflow execution information. This report includes running time, CPU and memory usage information as well as the job duration per process and sample.

## Results and discussion

We developed an open source bioinformatics tool that identifies the insertion site of a known transgenic sequence within a sufficiently contiguous host genome. The pipeline uses high-throughput data and takes advantage of discordant read pairs and chimeric reads. While it uses common bioinformatics software in the analysis of sequencing data, the different steps are collected in a main Nextflow script that simplifies its use. However, each step can be (re)run independently by modifying the configuration file, either to test different parameters without repeating previous steps or to troubleshoot. TC-hunter can analyze several samples simultaneously and automatically parallelize the processes to optimize the use of available computational resources. The complete output includes two user-friendly reports, one containing all predicted IS per sample with their corresponding graphical representations and the other showing performance statistics of each process.

### TIS identification in a transgenic mouse model using TC-hunter

Milosevic et al. (2021), established a transgenic mouse model overexpressing *PPM1D* by pronuclear injection to study the potential oncogenic properties of *PPM1D* [13]. Briefly, the transgenic construct harbors a rat TH promoter, a rabbit beta-globin intron and the human *PPM1D* cDNA, followed by an HSV terminator and the *AmpR* gene with its corresponding promoter (see Fig. 1 in [13]). To showcase the application of TC-hunter, four transgenic mice that harbored the human *PPM1D* gene and had the ability to pass this gene to their offspring were investigated. The identification of the TIS would aid in evaluating any potential phenotypic complications due to the random integration of the construct. The generation of these transgenic mice was carried out at Karolinska Center for Transgene Technologies (KCTT), with ethical approval numbers N251-12 and N42-14 [13], and were euthanized by carbon dioxide overdose followed by cervical dislocation. Three of these transgenic mice have been used in Milosevic et al. (2021).

WGS was performed in 4 transgenic mice DNA samples (Supplementary File 3), the resulting fastq files were analyzed with TC-hunter, using default parameters. In brief, a modified genome was created adding the transgenic construct sequence (9389 bp) to the mouse genome (GRCm38, GCA_000001635.6) as an extra chromosome. The TC-hunter configuration file was created and used to run the tool. The identification and visualization of TIS used minimal resources after the alignment step; each sample used an average of 1.85 CPUs with 3.68G of memory in ~ 13.45 min. Processing statistics, HTML reports and graphs can be found at https://github.com/bcfgothenburg/SSF. Merge with the previous paragraph The average read coverage over the host genome ranged between 33.48X and 59.53X (Supplementary Table S2).

### Output interpretation

**Fig. 2:**
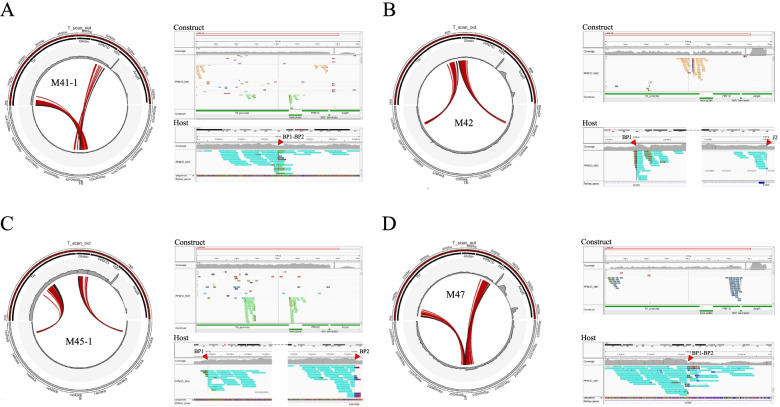
Transgenic insertion sites of four random integrations of a *PPM1D*-transgenic construct in mice as detected with TC-hunter. Results from TC-hunter of the best TIS for each sample analyzed (A-D). The circular representation shows the genomic region of the TIS in the host (bottom gray semicircle) and the genomic sequence of the transgenic construct (upper red semicircle). The different genomic features of the transgenic construct are depicted as black rectangles with their corresponding annotation. The histograms (in gray) show the sequencing coverage at the specified genomic regions. Discordant read pairs are shown as red lines, while chimeric reads are shown as black lines. The IGV snapshots show the supporting reads at the TIS in the construct (upper panel) and host (lower panel). Only chimeric reads are shown in both panels. These are grouped and colored by the chromosome of the mate; red arrows point to the validated breakpoints (BP1 and BP2) in the host. **A** TIS from sample 41. The *construct* panel shows the 2 predicted TIS. M41-1 corresponds to the best scoring TIS. M41-2 despite having a high score (8.011), could not be experimentally validated. **B** TIS from sample M42. Discordant pairs (in red) bisect the chimeric reads (in black) suggesting that the construct is inserted in the opposite direction with respect to the host. The TIS region spans over 29,531 bp. **C** TIS from sample M45. The TIS region span over 56,720 bp and the circular plot suggest a reverse insertion of the transgene. **D** TIS from M47. The transgene is also inserted in the opposite direction with respect to the host

TC-hunter identified a total of 8 TIS among the four samples with scores between 1.000 and 16.051. Sample M42 and M47 showed one predicted TIS each, while samples M41 and M45 presented two and four TIS candidates respectively (Table 1). Each candidate was manually inspected (see under *Filtering Strategy*), aided by the circular plots and IGV snapshots that TC-hunter automatically generated (Fig. 2). After removal of unreliable predictions four TIS, the best TIS candidate from each sample, were experimentally validated using Touchdown-polymerase chain reaction (TD-PCR) followed by Sanger sequencing (Supplementary File 3, Table S3 and Figure S1).

**Table 1:** TC-hunter summary report of all predicted transgene insertion sites across all the transgenic mice analyzed

**Sample**	**Host coverage** *average*	**Construct coverage** *average*	**TC-hunter score**	**Predicted breakpoint in host**	**Detected transgene region** *start—end*
M41	49.53	166.26	10.026	chr16:62,428,722–62,428,726	7﻿–6467^a^
			8.011	chr9:74,912,357–74,969,077	4649–2894*
M42	44.34	35.67	15.035	chr16:32,944,479–32,974,010	4289﻿–4129^a^
M45	49.40	260.81	16.051	chr9:74,912,357–74,969,077	4649﻿–2894^a^
			2.003	chr2:162,745,962–168,761,087	1994–7159*
			1.005	chr5:41,746,993–41,746,992	6509﻿–Unk*
			1.000	chr18:61,488,405–61,488,404	1442﻿–Unk*
M47	33.48	21.68	9.039	chr5:23,254,639–23,254,658	6537﻿–269^a^

^a^Validated breakpoints with Sanger sequencing

### Construct coverage

Sequencing data showed a similar coverage pattern of the construct among the four samples (Supplementary Figure S2). The rat TH promoter, the rabbit beta-globin intron and the human *PPM1D* cDNA had a consistent coverage, where the *AmpR* gene is unevenly covered.

Samples M42 and M47 had a read coverage over the construct of 44.34X and 33.48X, similar to the coverage over the host genome. However, the construct in sample M41 showed a coverage 3.4 times higher than the host genome, while for sample M45, the coverage was 5.3 times higher.

### Filtering strategy

There were two predicted TIS in sample M41. The top candidate was located in the intergenic region between *Gm33797* and *Nsun3* (chr16:62,428,722–62,428,726) with 26 discordant read pairs and 10 chimeric reads. The circular plot in Fig. 2A shows that discordant read pairs do not cross the corresponding chimeric read, i.e., looking at one predicted breakpoint, the red lines do not intersect any black line. This suggest that the construct is inserted in the same reading direction as the host which was confirmed by the experimental validation (Supplementary Table S3 and Figure S1). Figure S3 shows a decrease in the host coverage (66%) only for this sample, supporting the presence of a genomic rearrangement and therefore strengthening the reliability of the TIS prediction. Regarding the second candidate (9:74,912,357–74,969,077), although it presents a high score (8.011, Supplementary Figure S5 and Figure S7) together with an increase in coverage at the TIS (49.53X, Supplementary Figure S5 panel A) and the estimation of at least five copies of the *PPM1D* gene, it was not confirmed through PCR. This could be an example of a repetitive region being responsible of predicting a false positive TIS, since the primary hit is randomly selected from multimapping reads when no primary hit was initially found.

As for sample M42, TC-hunter predicted one single TIS (chr16:32,944,479–32,974,010) with 35 discordant read pairs and 15 chimeric reads. An interesting outcome is the large distance between the breakpoints of the TIS (29,531 bases). One of the breakpoints is located in the first intron of *Lrch3*, a gene involved in the regulation of actin in the cytoskeleton, while the other is in the seventh intron of the same gene. The duplication of the genomic material in the host (44%) and the estimated single copy of *PPM1D* may explain the large distance between the detected junctions (Supplementary Figure S4 and S8). The circular plot in 2B shows how the discordant read pairs cross the chimeric reads, i.e., looking at one predicted breakpoint, the red lines cut across the black lines. This suggest that the construct is inserted in the opposite direction with respect to the host. Indeed, the experimental validation supports that the construct is reverse inserted (Supplementary Table S3 and Figure S1).

For sample M45, four candidates were reported. The top candidate (9:74,912,357–74,969,077) with 51 discordant read pairs and 16 chimeric reads, also shows a large distance between the breakpoints (56,720 bases). One breakpoint is found in the intergenic region between *Onecut1* and *Fam214a* while the other is located in the second intron of *Fam21a*. There is a clear duplication event in the host genome (48% increase in coverage, Supplementary Figure S5) that together with the estimated 5 copies of *PPM1D*, may be indicative of several TIS or a tandem insertion of the transgene (Supplementary Figure S8). Similar to sample M42, the discordant read pairs suggest that the transgene is reversed inserted in the host (Fig. 2C). The remaining candidates had a low score (less than 2.003) which may be indicative of false positive predictions. Moreover, looking through their circular plots this may reassure their identification as false positives hits (Supplementary Figure S7). For instance, M45-3 and M45-4 are supported only by one predicted junction, while the discordant read pairs in M45-2 does not show a convincing pattern. In addition, scanning the coverage over the TIS, there is no evidence of any genomic rearrangement (Supplementary Figure S5 panels B-D). However, the presence of multiple copies of the *PPM1D* gene may be indicative of several TIS where only experimental validation may truly distinguish between real and uncertain predictions. In this case, these three TIS were disregarded as false predictions.

Finally, for sample M47, there was only one predicted TIS (chr5:23,254,639–23,254,658) located in the third intron of *Lhfpl3*, a gene associated with deafness in humans and mice, with 39 discordant read pairs and 9 chimeric reads giving support to the breakpoint. The circular plot in Fig. 2D suggests that the construct is inserted in the opposite direction with respect to the host, which was confirmed by the experimental validation (Supplementary Table S3 and Figure S1). Another supporting genomic rearrangement is the sudden decrease in coverage (44%) over the TIS (Supplementary Figure S6).

### Considerations of transgene detection

There are some common considerations in the identification of TIS including dealing with poorly assembled genomes, repetitive regions and the possibility of having multiple IS in one sample. TC-hunter can satisfactorily identify TIS in genomes that are properly assembled such as those of *Mus musculus, Drosophila melanogaster, Glycine max and Oryza sativa*, examples presented in this work. However, for poorly assembled genomes, i.e. highly fragmented genomes, TC-hunter might miss to identify a TIS if the IS are located in different contigs since the current version assumes the breakpoints to collocate in the same DNA fragment.

Genomes harbor thousands of copies of very similar transposable elements sequences and when these regions are sequenced and aligned, the resulting reads map to several locations [24]. In the current version of TC-hunter, these multimapping reads may be a source of false positive hits since one single location is randomly selected when no primary hit is initially found in the mapping step by BWA. However, these hits may be filtered away when inspecting the circular graphs and supporting data (Supplementary Figure S7).

Lastly, the use of lentivirus transgenesis often generates transgenic organisms with multiple integration events and random TIS [25]. At the moment, TC-hunter does not distinguish between multiple IS within the same scaffold. Nonetheless, inspecting the graphical aids, the chimeric reads and discordant read pairs may hint of these multiple events. We expect to implement solutions to these pitfalls in future versions of TC-hunter.

### Performance evaluation with simulated data

**Fig. 3:**
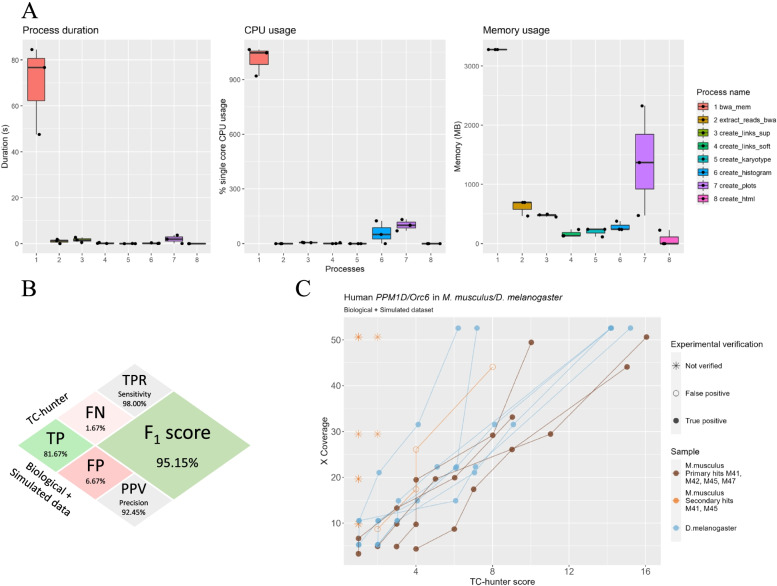
TC-hunter performance evaluation and detection level. **A** Time and resources consumption of TC-hunter using simulated data (human *Orc6* in *D. melanogaster*), every step in the pipeline is illustrated. **B** Confusion matrix showing the performance of TC-hunter for 35 samples (simulated and biological data). TP, true positive; FP, false positive; FN, false negative; TPR, true positive rate; PPV, positive prediction value and F_1_-score, harmonic mean of precision vs sensitivity. **C** Correlation between average coverage of a sample and the score from the predicted TIS. TIS from simulated data are shown in blue. TIS from real biological data are shown in brown (best candidate) and orange (secondary predictions). True positive predictions are shown as filled circles, false positives are open circles and predictions that were not experimentally validated are shown as asterisks and thus, not joined with a line

To evaluate the performance of the pipeline, three whole genome sequencing (WGS) datasets from the *Drosophila melanogaster* genome (dm6, GCA_000001215.4) were simulated, where the human gene *Orc6* (NC_000016.10) was inserted at several known positions with an average coverage of approximately 50X. Since genomic deletions have been seen to cooccur at the TIS in the host genome [26–28], some coincident deletions were included in the simulation datasets (Supplementary File 3 and Table S2). All samples were analyzed with TC-hunter using default parameters. The Nextflow workflow reports can be accessed at https://github.com/bcfgothenburg/SSF. The average running time for each sample was 1 h and 5 min, where approximately 90% of the time was spent on the mapping step with a CPU usage of 10 cores at 100% and the rest of the steps used 1.7 cores in average (Fig. 3A). In terms of memory usage, the entire process averaged to 6.34G. A third of the memory was used in the creation of visual aids while half of the memory usage was devoted to the identification of the transgene and its insertion site.

To investigate the performance of TC-hunter with low coverage data, the *Drosophila melanogaster* simulated datasets and the transgenic mice samples were downsampled (Supplementary File 3). A total of 35 samples harboring 60 TIS were analyzed by TC-hunter obtaining an overall good performance (sensitivity = 98.00% and precision = 92.45%), identifying 81.67% of the true TIS events from the simulated and the real datasets (Fig. 3B).

All TIS were correctly identified in the simulated data, except for one TIS in a sample with low coverage (5X). In the real dataset, TC-hunter predicted all verified IS at any given coverage and suggested 13 secondary hits at coverages higher than 5X (Supplementary Table S2). After closer inspection, these hits were either false positives or were not verified experimentally (Fig. 3C).

### Comparison with other tools

To benchmark TC-hunter against other algorithms an exhaustive literature review was performed (Supplementary Table S1). The PubMed database was queried using *(transgene insertion sites identification) AND (bioinformatics)* (last accessed 02–06-2021). Nine publications were found and all references within, that described any kind of algorithm or program related to the identification of IS, were retrieved. A total of 31 algorithms were found, from which ten pipelines focused on the identification of retrotransposable and transposable elements, while sixteen dealt with viral and vector integrations. Only five pipelines explicitly targeted the identification of TIS using high-throughput data. These pipelines rely on discordant read pairs and chimeric reads. CONTRAILS [10] is a pipeline that generates a reference genome sequence adding the transgene sequence as an extra chromosome and after alignment, BLAST [29] is used on the discordant pairs to further characterize the insertion site. Srivastava et al. (2014) applies a scoring system, where the genome is divided into blocks and the number of mapped reads is determined. This is then compared to a genome-wide threshold to calculate the significance of the TIS. The workflow is available as a collection of perl scripts and consists of eight detached steps. Among the in-house tools, Zhang et al. (2012) uses de novo assemblies from unmapped reads and the resulting contig(s) are blasted against the transgene. Chimeric reads and discordant read pairs are then used as supporting evidence. Similarly, Abrams et al. (2014) uses chimeric reads to identify the insertion site, while the discordant read pairs are used to identify structural variants with third party tools. Lastly, transgeneR [12] is an R package that applies a two-round alignment, it relies on split reads and coverage to identify the TIS and calculates the confidence of the call.

Unfortunately, three of them [8, 10, 11] were not publicly available at the time we performed the literature review. The other two pipelines, Srivastava et al. (2014) and transgeneR, were installed and tested (Supplementary File 3). Although the documentation from Srivastava et al. (2014) is simple and easy to follow, no results were obtained after analyzing our samples with the default settings. We scrutinized the intermediate files and despite considering that the correct chimeric reads were recovered, no candidates were suggested after the calculation of the window score. Regarding transgeneR [12], the installation was not properly tested given that only the results of the test dataset were provided within the documentation. While running the package, some errors were encountered preventing the analysis to properly finalize, thus no TIS were reported. The lack of test data, code documentation and response from the author, restrained us from using the tool. We consider that publicly available tools should not require extensive troubleshooting and that proper documentation must be accessible and complete. This will redirect the technical efforts towards the biological interpretation of the results. On this note, we have ensured that TC-hunter is easy to install and use. Step by step instructions are available in GitHub and its installation is done via a conda environment file, which can be tested with the accompanying dataset.

Given the absence of functional and available tools, we scanned the five TIS prediction software described in Supplementary Table S1 for suitable datasets to analyze with TC-hunter. We downloaded the data from Lambirth et al. (2015) and an extra dataset from Yang et al. (2013) (Supplementary Table S2), all other references were either missing the construct sequence (or incorrect), or the sequencing data was not available. The soy sample ST77-KP2, harbors the human thyroglobulin gene (hGT) [10]. For the rice samples, being a co-transformed insect-resistant rice strains, T1c-19 includes the *bar* gene, an herbicide resistance gene [30], while TT51 harbors a fused *Bt* insecticidal gene and the hygromycin resistant gene *hpg* [31, 32]. These three samples were analyzed with TC-hunter (default settings). The soy reference (Wm82.gnm4.4PTR) and the rice reference (OSchrV7) were downloaded from soybase.org and plangdb.org, respectively. The ST77 construct sequence was downloaded from https://bitbucket.org/lorainelab/soyseq/src/master/InsertionAnalysis/data/snapGene while the rice construct sequences were obtained from [30] (Supplemental sequence No. 1).

We were able to identify the same soy TIS (Supplementary Table S2 and Figure S9) as described in Lambirth et al. (2015). Yang et al. (2013) detected two TIS in T1c-19 and three TIS in TT51, TC-hunter failed to detect one TIS in each sample, likely due to a repetitive region in chr04. An interesting phenomenon involves the *actin* promoter, which is present in the rice genome as well as the construct, leading to reads mapping to at least two regions. In sample TT51, there are 2 candidates, one in chr3 (score = 7.083) and the other in chr10 (score = 2.04). Intuitively, the best candidate would have the higher score, however in this case, Lambirth et al. (2015) experimentally verified the secondary hit as a true positive TIS, while the one with the higher score was a false positive hit. This exemplifies the importance of inspecting all candidates despite their low score.

## Conclusions

Identifying the insertion site of a transgene is crucial to evaluate any possible disruption in the desired phenotype while generating transgenic animals. Current bioinformatic tools using high-throughput data designed for this purpose are either not publicly available or not properly maintained. In this study we presented TC-hunter, an open bioinformatics tool that identifies transgene insertion sites from DNA sequencing data. We demonstrated its application by identifying and experimentally validating the transgenic insertion sites of the human *PPM1D*, a p53-regulated gene, in a transgenic mouse model used in the study of cancerogenesis.

We believe that TC-hunter will be a valuable tool since it generates user friendly reports and visualizations that aid in understanding the genomic rearrangements that could compromise the expected phenotype.

To further improve the value of TC-hunter, a user-friendly web application may appeal to researchers and clinicians who prefer point and click tools. In addition, due its modular implementation, functionalities such as the automatic identification of contiguous TIS or the detection of construct-to-construct fusions may be easily implemented in future versions of TC-hunter.

## Supplementary Information

**Additional file 1.** Supplementary Figures.

**Additional file 2.** Supplementary Tables.

**Additional file 3.** Supplementary Methods.

## Data Availability

Whole genome sequencing data of the four transgenic mice generated and analyzed during the current study are available at the NCBI Sequence Read Archive (SRR17772243, SRR17772244, SRR17772245, SRR17772246) with BioProject reference PRJNA662713.

## Abbreviations

IGV: Integrative Genomics Viewer
IS: Insertion site
PCR: Polymerase Chain Reaction
TC-hunter: Transgene-Construct hunter
TD-PCR: Touchdown-polymerase chain reaction
TIS: Transgene insertion site
NGS: Next generation sequencing
WGS: Whole genome sequencing
