# Supplementary material for: TC-hunter: identification of the insertion site of a transgenic gene within the host genome
Source: BMC Genomics. 2022 Feb 20;23:149. doi: 10.1186/s12864-022-08376-0 (PMC8859905; doi:10.1186/s12864-022-08376-0)
Supplement: Supplementary file 1 — Additional file 1. Supplementary Figures. [file 12864_2022_8376_MOESM1_ESM.pdf]

Additional File 1. Supplementary Figures

TC-hunter: Identification of the insertion site of a transgenic gene within the host genome

Vanja Börjesson[1], Angela Martinez-Monleon[2], Susanne Fransson[2], Per Kogner[3], John Inge Johnsen[3], Jelena Milosevic[3,4] and Marcela Dávila López[1,*]
[1] Bioinformatics Core Facility, Sahlgrenska Academy, University of Gothenburg, Sweden.
[2] Department of Laboratory Medicine, Institute of Biomedicine, Sahlgrenska Academy, University of Gothenburg, Gothenburg, Sweden.
[3] Childhood Cancer Research Unit, Department of Women's and Children's Health, Karolinska Institutet, Stockholm, Sweden.
[4] Center for Regenerative Medicine, Massachusetts General Hospital, Boston, MA 02114, USA.

Figure S1. Experimental validation of TIS.

Figure S2. Sequence coverage over the human *PPMD1* construct.

Figure S3. Sequence coverage over the best TIS in sample M41.

Figure S4. Sequence coverage over the sole TIS in sample M42.

Figure S5. Sequence coverage over the four TIS in sample M45.

Figure S6. Sequence coverage over the sole TIS in sample M47.

Figure S7. Circular plots of secondary TIS predictions.

Figure S8. Suggested model describing a transgenic insertion in a duplicated genomic region in the host.

Figure S9. Circular plots of TIS predictions in *G. max* and *O. sativa*.

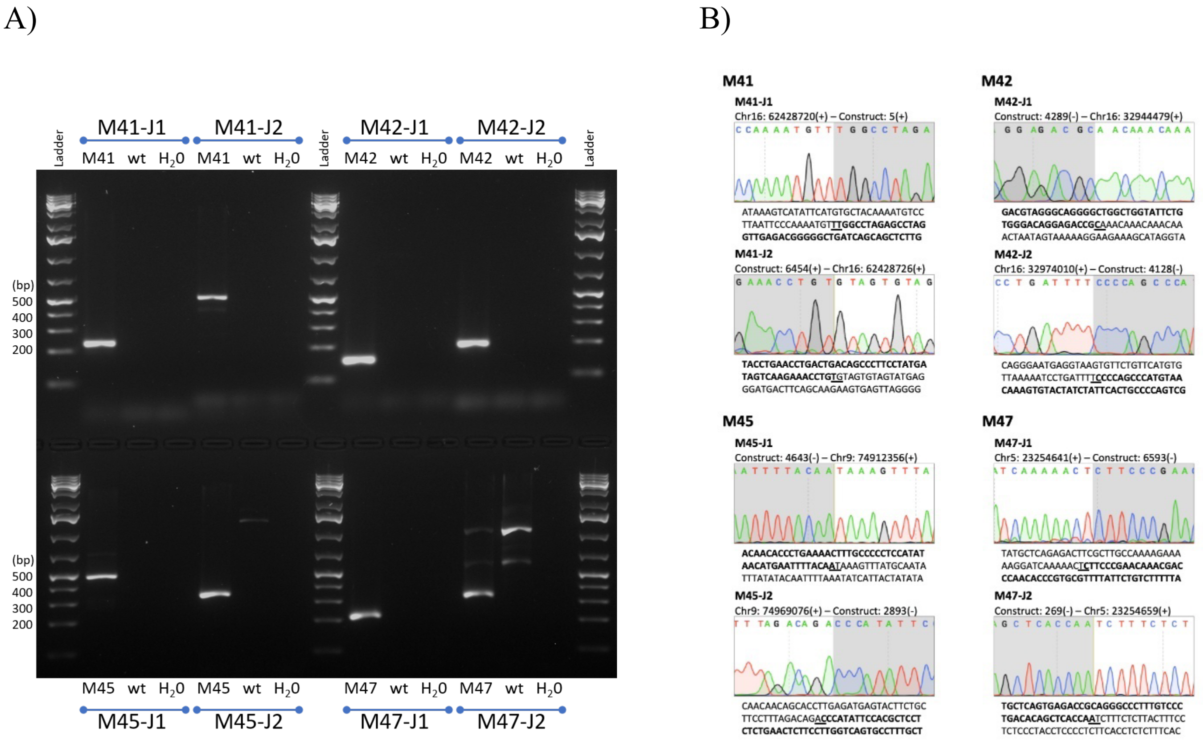

Figure S1. Experimental validation of TIS. A) Electrophoresis of PCR products detected through Touchdown-polymerase chain reaction (TD-PCR). **B)** Sanger sequencing chromatograms of the validated junction sites.

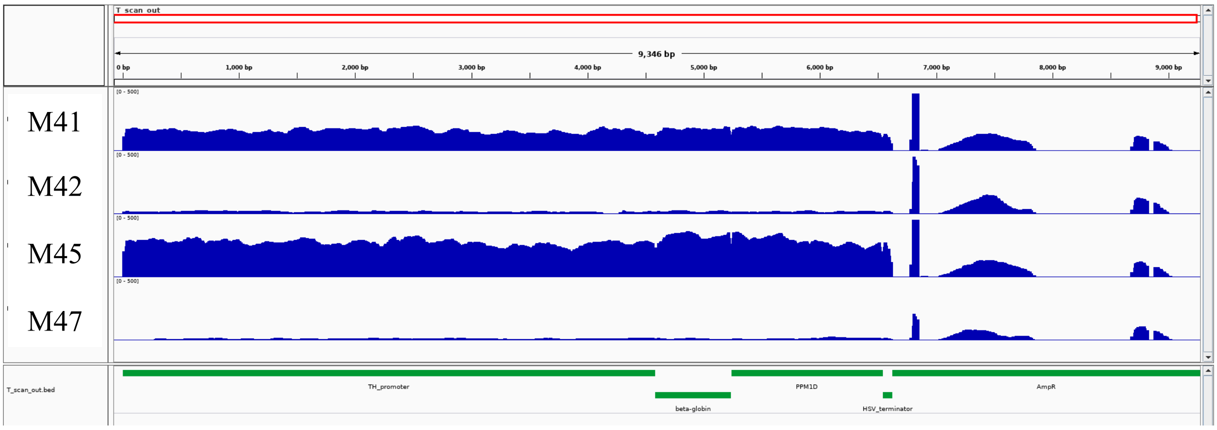

Figure S2. Sequence coverage over the human *PPMD1* construct. The different genomic elements of the construct are depicted as green rectangles at the bottom of the figure.

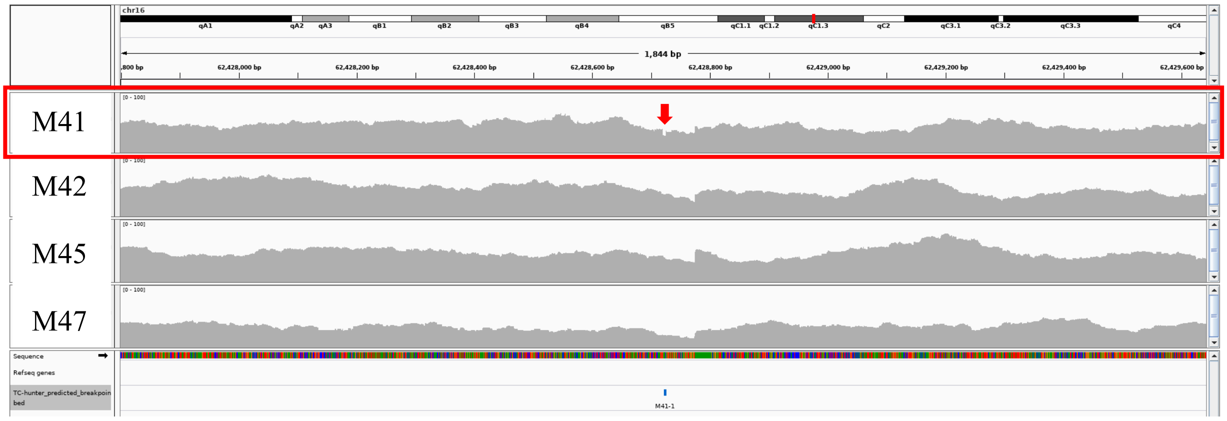

Figure S3. Sequence coverage over the best TIS in sample M41. Red arrow points to a decrease in coverage due to the IS of the transgene. Not seen in other samples. TIS predicted by TC-hunter is shown as a blue rectangle at the bottom of the figure. For the secondary candidate, M41-2, refer to Figure S5.

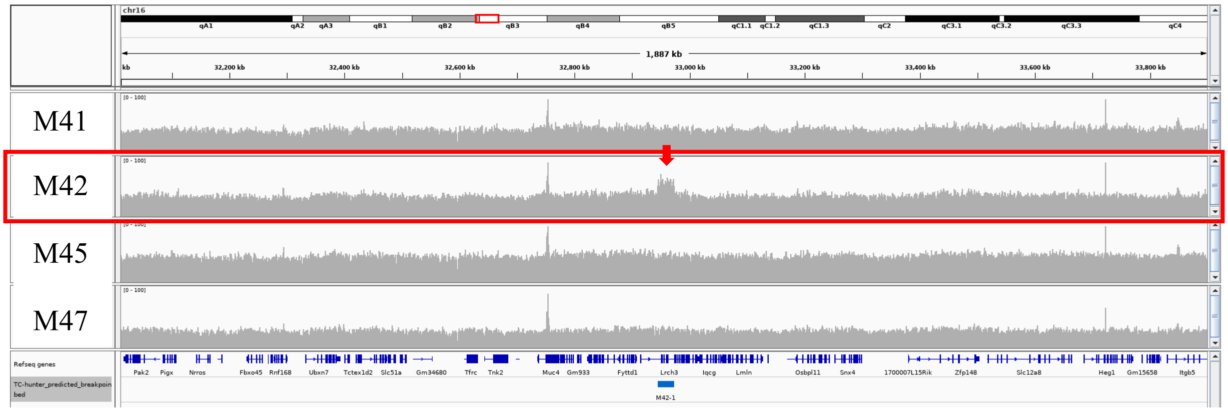

Figure S4. Sequence coverage over the sole TIS in sample M42. Red arrow points to an increase in coverage due to the IS of the transgene. Not seen in other samples. TIS predicted by TC-hunter is shown as a blue rectangle at the bottom of the figure.

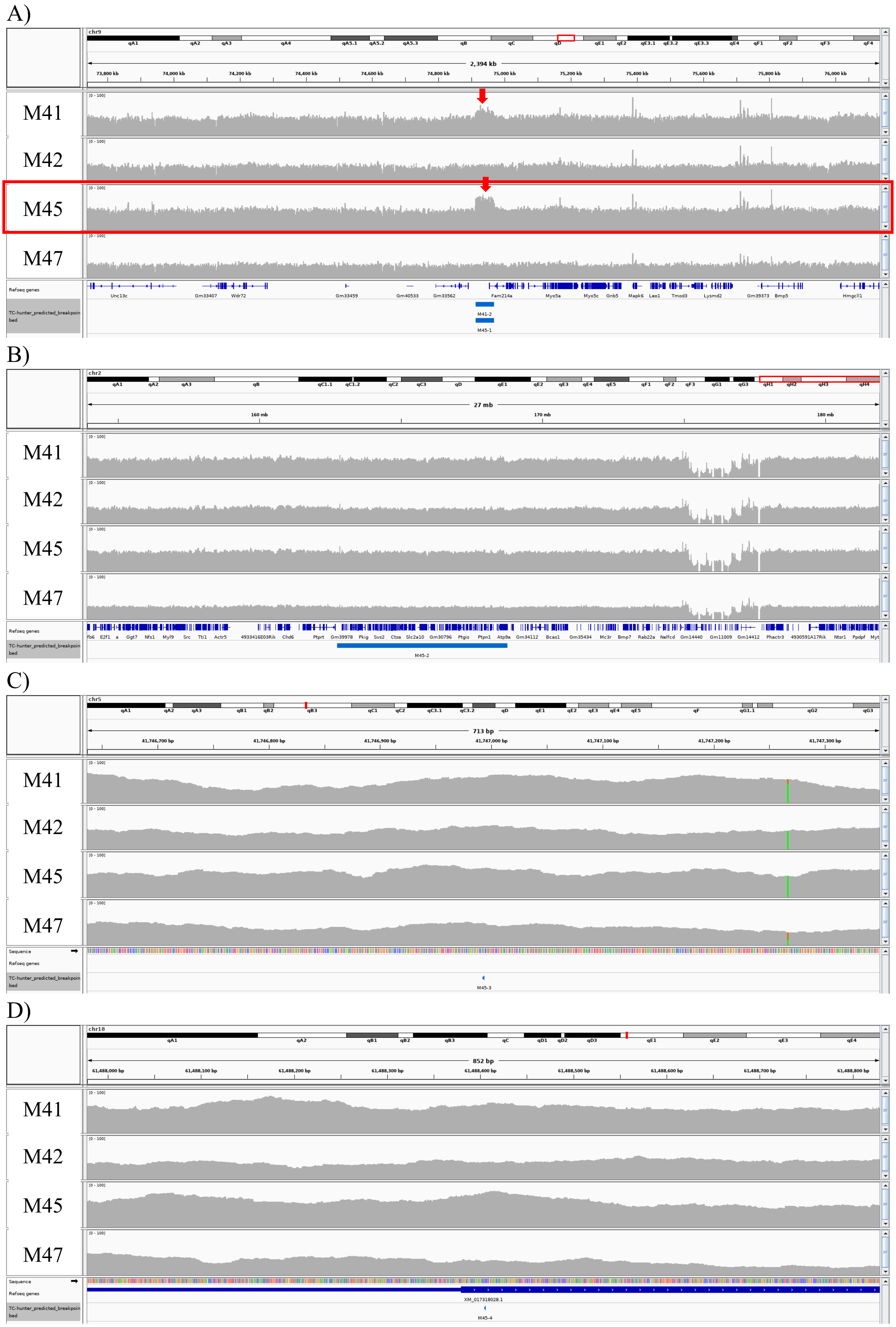

Figure S5. Sequence coverage over the four TIS in sample M45. (A-D) ordered by score. Red arrow points to an increase in coverage due to the IS of the transgene. A similar, but minor increase is seen in sample M41. TIS predicted by TC-hunter is shown as a blue rectangle at the bottom of the figure. See under Discussion in the main text.

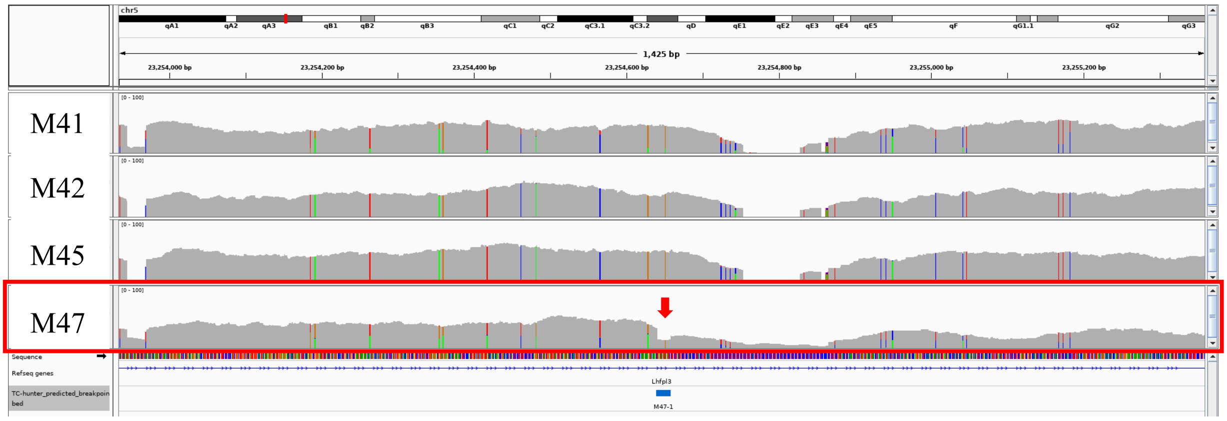

Figure S6. Sequence coverage over the sole TIS in sample M47. Red arrow points to a decrease in coverage due to the IS of the transgene. Not seen in other samples. TIS predicted by TC-hunter is shown as a blue rectangle at the bottom of the figure.

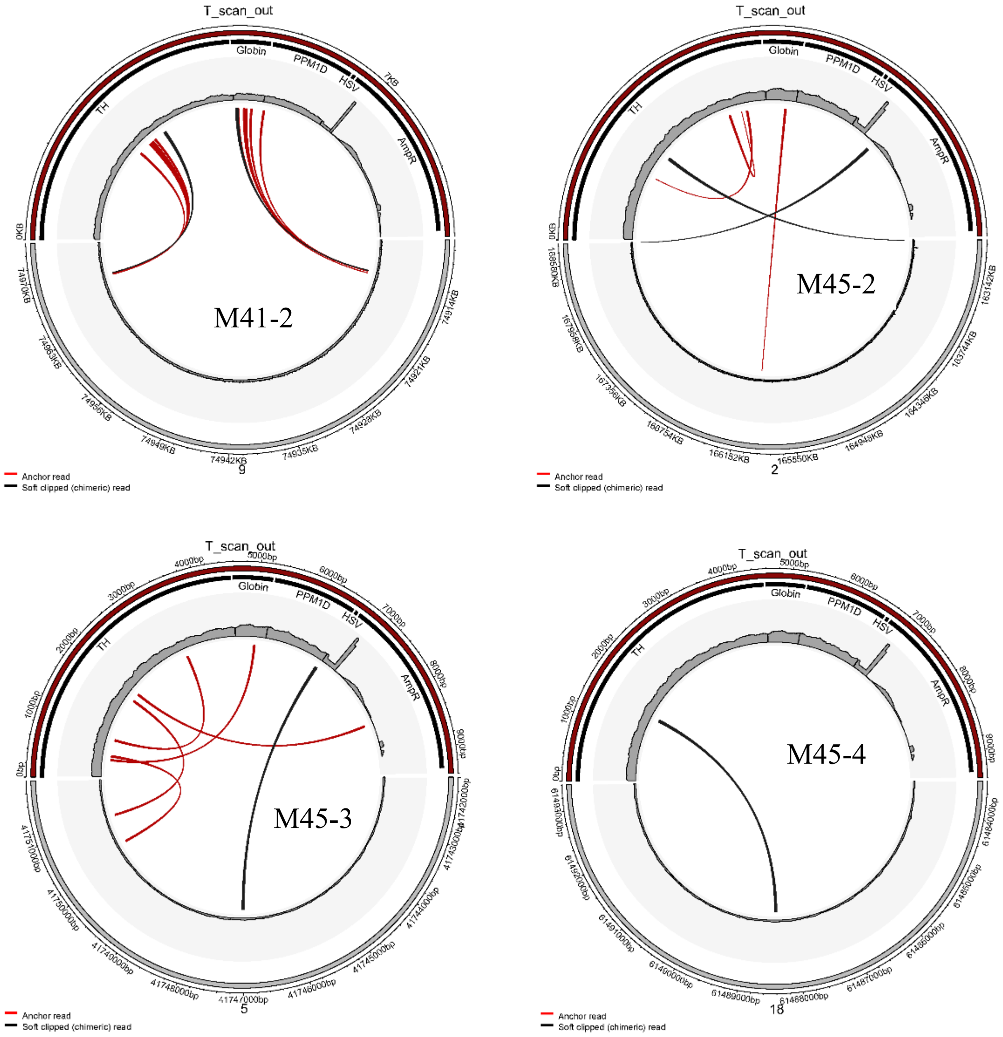

Figure S7. Circular plots of secondary TIS predictions. The genomic region of the TIS in the host (bottom gray semicircle) and the genomic sequence of the transgenic construct (upper red semicircle) are depicted. The different genomic features of the transgenic construct are depicted as black rectangles with their corresponding annotation. The histograms (in gray) show the sequencing coverage at the specified genomic regions. Discordant read pairs are shown in as read lines, while chimeric reads are shown as black lines.

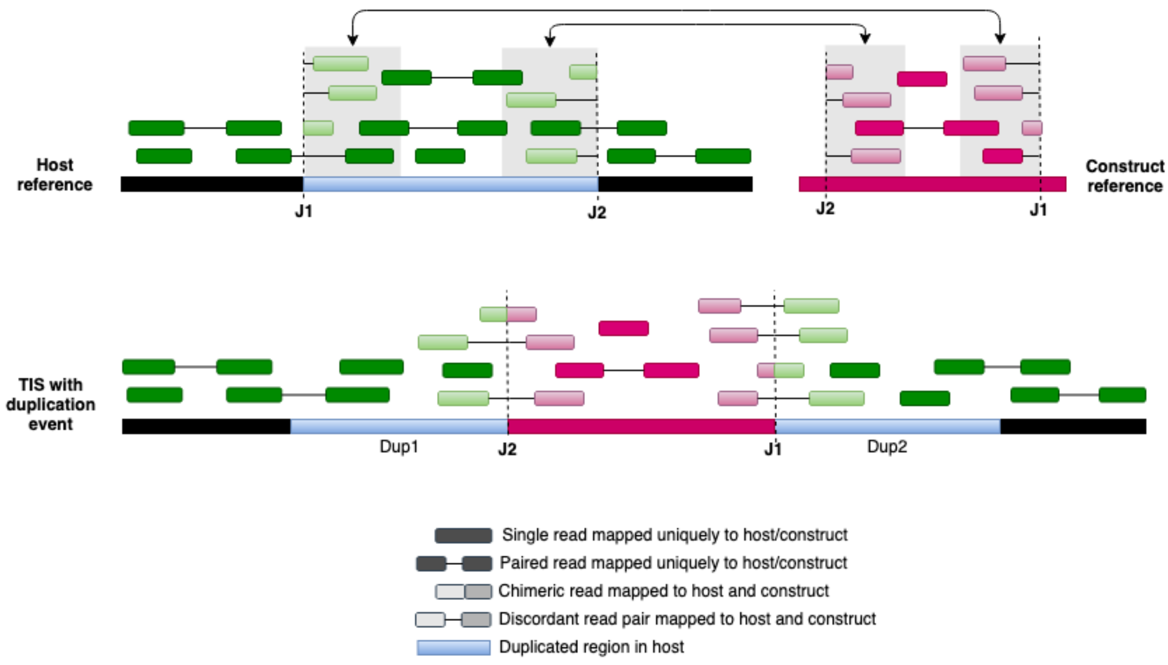

Figure S8. Suggested model describing a transgenic insertion in a duplicated genomic region in the host. Possible genomic rearrangement at the TIS in samples M42 and M45 according to the coverage data. *Upper panel*: Discordant and chimeric reads mapped to host pointing outwards the insertion site (light rectangles) and the read coverage between junction sites (J1 and J2) clearly exceeding the average coverage, indicates a possible duplication event at the TIS. *Lower panel*: Theoretical arrangement of duplicated segments at the TIS in the sample.

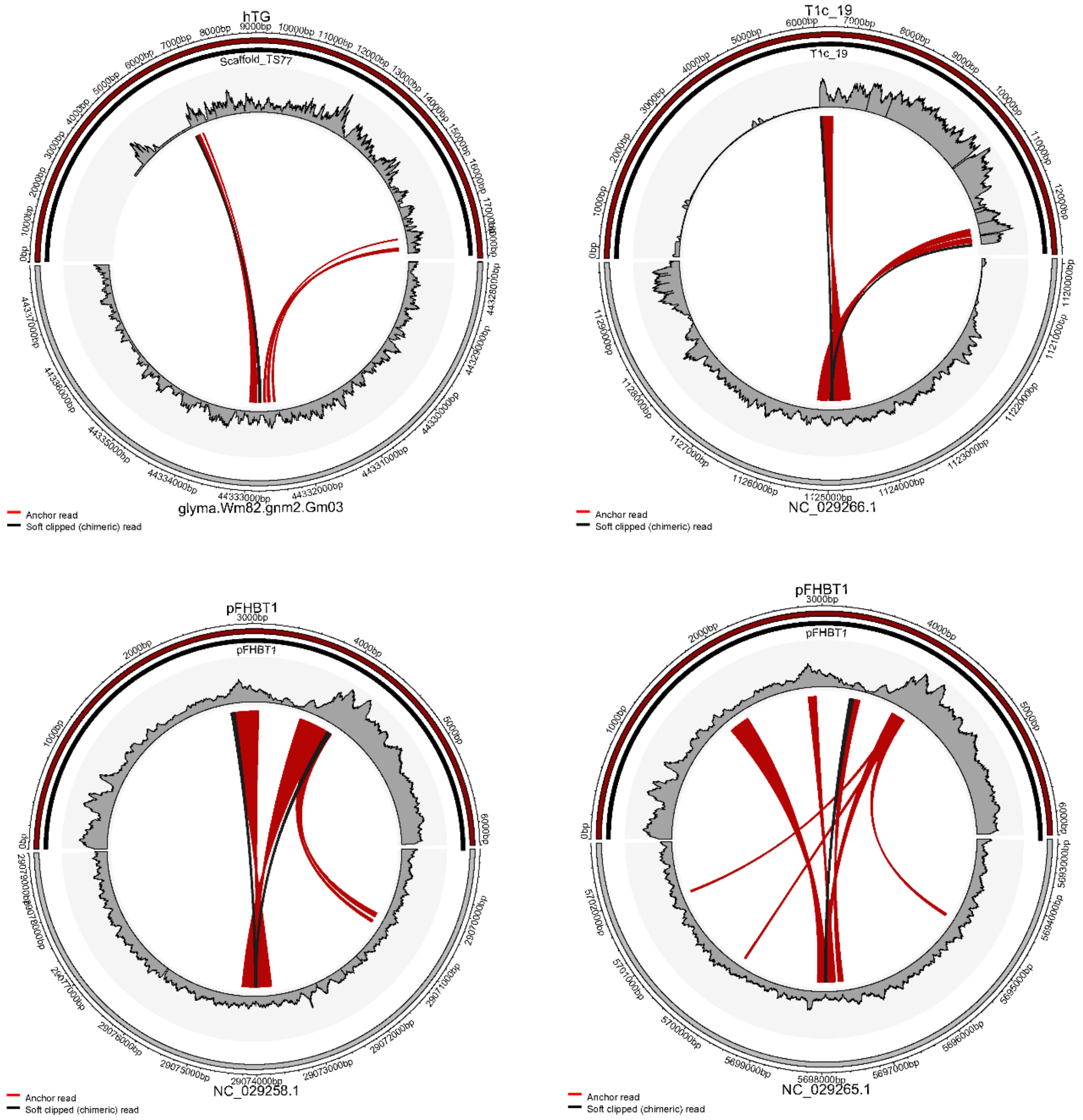

Figure S9. Circular plots of TIS predictions in *G. max* and *O. sativa*. Visualization of the predicted TIS by TC-hunter. Legend as in Figure S7.
