# Supplementary material for: TC-hunter: identification of the insertion site of a transgenic gene within the host genome
Source: BMC Genomics. 2022 Feb 20;23:149. doi: 10.1186/s12864-022-08376-0 (PMC8859905; doi:10.1186/s12864-022-08376-0)
Supplement: Supplementary file 3 — Additional file 3. Supplementary Methods. [file 12864_2022_8376_MOESM3_ESM.pdf]

Additional File 3. Supplementary Methods

TC-hunter: Identification of the insertion site of a transgenic gene within the host genome

Vanja Börjesson[1], Angela Martinez-Monleon[2], Susanne Fransson[2], Per Kogner[3], John Inge Johnsen[3], Jelena Milosevic[3,4] and Marcela Dávila López[1,*]

[1] Bioinformatics Core Facility, Sahlgrenska Academy, University of Gothenburg, Sweden.

[2] Department of Laboratory Medicine, Institute of Biomedicine, Sahlgrenska Academy, University of Gothenburg, Gothenburg, Sweden.

[3] Childhood Cancer Research Unit, Department of Women's and Children's Health, Karolinska Institutet, Stockholm, Sweden.

[4] Center for Regenerative Medicine, Massachusetts General Hospital, Boston, MA 02114, USA.

Whole Genome Sequencing DNA

Total DNA was extracted from tails of the four transgenic mouse strains using the Quick-DNA Miniprep Plus Kit, D4068, Zymo Research. Sequencing libraries were prepared using 8-12 µg DNA per mouse and the Illumina TruSeq PCR-free kit according to the manufacturer's protocols. Cluster amplification was done by cBot and samples were sequenced on HiSeqX (HiSeq Control Software HD 3.4.0.38/RTA 2.7.7) with a 2x151 setup using HiSeq X SBS chemistry. Between 616M and 909M reads were generate (Supplementary Table S2). All raw sequence reads were quality controlled using FastQC (Babraham Bioinformatics, Cambridge, United Kingdom).

Touchdown-polymerase chain reaction (TD-PCR)

To validate the junctions detected by TC-hunter, specific primers for the breakpoints of the different construct/mouse DNA junctions were designed (Supplementary Table S3) using the sequencing information previously obtained.

The TD-PCR was performed using the Platinum PCR Master Mix from Invitrogen (12358-010) under standard conditions and the specific primer for the breakpoints. In the case of M47-J2 a SuperFi GC Enhance (provided together with the master mix) was added due to the high GC content in the primers.

The TD-PCR conditions were the following: pre-denaturation 95°C (2 min), 20 cycles of denaturation 98°C (10 sec), annealing 68°C (10 sec) were the temperature was gradually reduced in -0,5°C per cycle and extension 72°C (15 sec) followed by other 20 cycles of denaturation 98°C (10 sec), annealing 58°C (10 sec) and extension 72°C (15 sec) with a final extension at 72°C (1min), holding at 4°C.

The electrophoresis of the PCR products was performed in a 1,5% Agarose gel (Thermo scientific, R0491) in TAE (1X) buffer (Invitrogen, 15558-026) using GelRed as a gel stain (biotium, 41003) and GeneRuler 1kb plus as a ladder (Thermo scientific, SM1331).

Sanger sequencing

The amplicons generated by the TD-PCR were Sanger sequenced by Eurofins Genomics under "Supreme Run" conditions, using the specific primers previously mentioned (Supplementary Table S3). The chromatogram traces of the sanger sequencing were visualized using SnapGene Viewer (from Insightful Science; available at snapgene.com). To verify the sequences, the results obtained were compared to the DNA of the mouse and the construct using BLAT [25] and BLAST [15] respectively.

Data simulation

To validate the pipeline, two simulated transgenic genomes and one negative control were generated. The human gene *Orc6* (NC_000016.10) was used as transgene and was inserted in the *Drosophila melanogaster* (dm6) genome at various positions. Deletions at different position were also simulated by randomly removing sections of the selected fastq files. See Supplementary Table S2 for the exact number of simulated reads and the exact positions of the simulated deletions. wgsim [24] was used to generate around 40M pair-end reads (50x coverage) for all three genomes setting the read length to 90 bp, outer distance between pairs to 500 bp and the mean coverage across positions to 50x while no mutations were introduced.

Data downsampling

To evaluate the ability of TC-hunter to predict transgenic insertion sites with low read coverage, we used samtools [20] to downsample the aligned datasets. The *D. melanogaster* simulated data (50X) was downsampled to to 30X, 20X, 10X and 10X and 5X as mean read coverage (Supplementary Table S2). The *M. musculus* transgenic samples were downsampled to 60%, 40%, 20%, 10% and 5% of the total reads. See Supplementary Table S2 for exact number of reads.

Transgenic Insertion Site analysis with other tools

The TIS scoring procedure from Srivastava et al [9] and transgeneR [12], were installed following the instructions provided at their corresponding publications. The first step of the eight processes in the TIS scoring procedure was omitted. This step refers to the filtering and quality control of the data which was previously done. The remaining seven steps were run without any warnings or errors. The transgeneR package was run following its documentation despite of not being able to assess the installation (the test dataset was unavailable). Unfortunately, the analysis failed and despite our best efforts, the lack of documentation and support hindered us from successfully running the tool.

TC-hunter analysis on external datasets

To evaluate the performance of TC-hunter on other biological samples, we analyzed the soy sample ST77-KP2 from [10] and the two rice samples T1c-19 and TT51 from [30]. The sequencing data was downloaded from NCBI Sequence Read Archive (SRA) using accession number SRR2180176 and SRR567751, construct sequences was retrieved from the papers, and host reference genomes used was William 82 genome assembly v.4.0 for soybean sample and Oryza sativa Japonica Group assembly IRGSP-1.0 for rice samples. The rice reference differs from the reference used in the paper, and a manual translation of the breakpoint positions was done using BLAST [29] to make them comparable. See Supplementary Table S2 for verified TIS. TC-hunter was run using default parameters except for mapping quality threshold that was set to 10.
